# Engineering versatile supramolecular structures with tetravalent DNA-traptavidin building blocks

**DOI:** 10.1039/d5na00575b

**Published:** 2025-09-02

**Authors:** Dayoung Gloria Lee, Young-Youb Kim, Hoonil Yang, Yoon-Kyu Song

**Affiliations:** a Department of Applied Bioengineering, Graduate School of Convergence Science and Technology, Seoul National University Seoul 08826 Republic of Korea songyk@snu.ac.kr; b Research Institute for Convergence Science, Seoul National University Seoul 08826 Republic of Korea; c Department of Chemical Engineering, Columbia University New York New York 10027 USA

## Abstract

Proteins offer great promise for nanoscale biomaterials, but precise assembly is challenging. This study presents DNA-protein hybrid building blocks (DTHBs) using traptavidin and biotinylated DNA for controlled assembly to linear chains, spherical clusters, and ordered lattices, enabling sophisticated, programmable architectures with potential biomedical applications, including drug delivery and biosensors.

## Abstract 2

Programmable DNA-protein building blocks were created by attaching DNA strands to traptavidin, enabling the directed self-assembly of supramolecular structures, such as chains, clusters, and lattices, through sequence-specific DNA hybridization.

## Graphical abstract

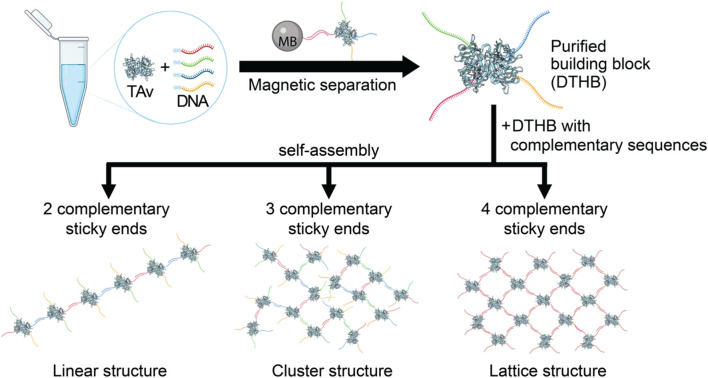

Proteins are promising nanoscale building blocks due to their unique properties and structural diversity.^1–3^ Self-assembling proteins into well-organized nano-to mesoscale structures can lead to innovative biomaterials with applications in biology. However, the complex surfaces of proteins pose challenges for engineering sophisticated structures with precise positioning.^4,5^ Recent advancements in DNA nanotechnology have facilitated the creation of DNA-protein hybrid building blocks, where DNA serves as a template to anchor and direct assembly.^6–8^ DNA's programmability through Watson-Crick base pairing provides specific connectivity and binding orientation, combining the biological functions of proteins with the programmability of DNA.^9,10^ This approach offers a means to achieve precisely programmed assembly while overcoming the limitations posed by the unpredictable structures of protein assemblies.

A key strategy in developing DNA-protein hybrid building blocks is the site-specific conjugation of DNA to proteins in a precise stoichiometric manner.^11,12^ By attaching DNA to a protein, we can create a versatile genetic code with four nucleobases (A, T, G, C), enabling the engineering of supramolecular assemblies into defined spacings and diverse morphologies, from one-dimensional (1D) to three-dimensional (3D) architectures. Traditional DNA-to-protein conjugation methods often rely on chemical reactions that modify specific amino acid residues,^13^ resulting in heterogeneous products with varying DNA-to-protein stoichiometries and unwanted modifications, leading to unpredictable assembly behavior.^14^

To achieve the desired DNA-protein conjugation without post-chemical modification, we utilized the avidin-biotin interaction, which is known for the strongest non-covalent interaction between protein and ligand.^15^ In this study, we utilize traptavidin (TAv) as the core protein, which is a genetically modified streptavidin variant developed for thermal stability and enhanced biotin-binding stability. We present a platform for constructing supramolecular assemblies using DNA-TAv conjugates. By varying the DNA sequences attached to TAv, we program the assembly of diverse structures ranging from simple 1D chains to complex 3D lattices.^16,17^ We introduce the DNA-TAv hybrid building block (DTHB), which enables the assembly of various supramolecular structures through a straightforward programming approach (Fig. 1).

**Fig. 1:**
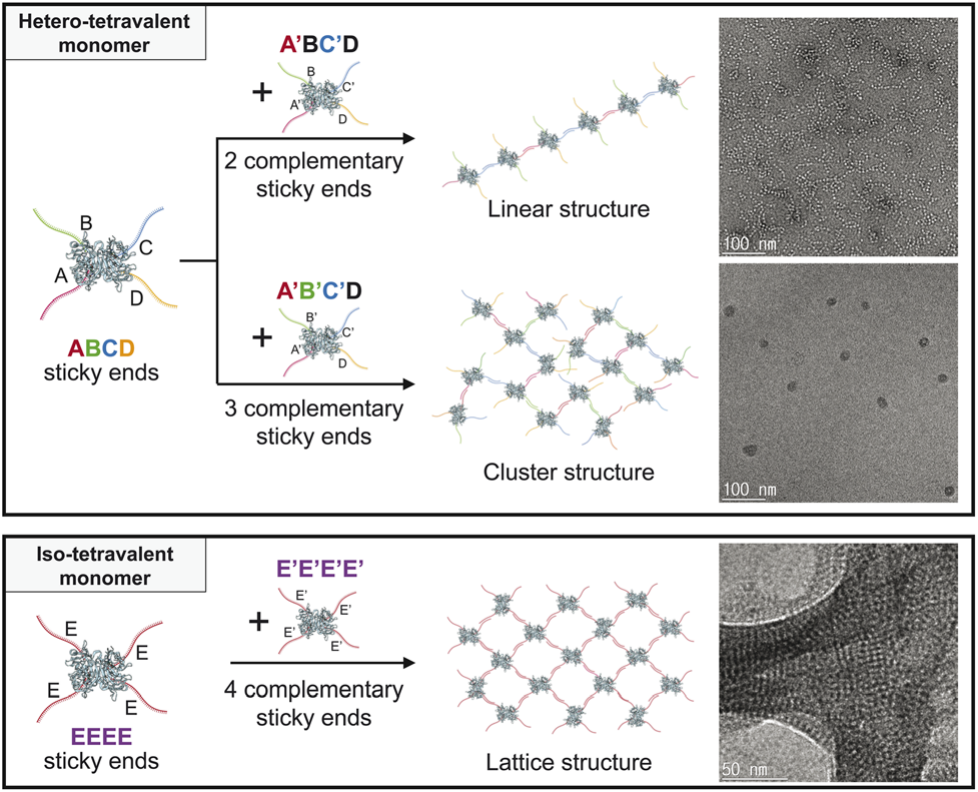
Schematic illustration of the programming supramolecular nanostructure assembly using hetero- and iso-multivalent monomers through varying the number of complementary DNA oligomer linkers in DTHBs.

TAv is a tetrameric protein, which has four biotin binding sites; thus, mixing TAv with four different biotinylated DNA strands can create tetravalent DNA-protein building blocks. However, when we introduce multiple DNA sequences, various building blocks will be created due to numerous possible permutations, as shown in Fig. S1. Therefore, a specific building block selection protocol is crucial to direct complex assembly. In this study, we utilized a stepwise magnetic separation technique adapted from our group's previous paper to synthesize heterotetravalent DTHBs with four unique DNA sequences^18,19^ (Fig. S1). TAv shows a significant decrease in biotin dissociation rate, increased thermal stability, and stronger mechanical binding strengths compared to streptavidin,^20^ ensuring sample reliability and yield during multiple steps of magnetic separation, as demonstrated in previous studies.^18,19^ Repeated magnetic separation steps purified DNA-conjugated building blocks to distinct tetravalent DTHBs, allowing sophisticated supramolecular architectures to be assembled (Fig. S2).

We fabricated well-defined supramolecular architectures by mixing two types of DTHBs for self-assembly into programmable structures, such as linear, cluster, and lattice structures, through a simple mixing and slow cooling process. The assembly was programmed by designing the number of complementary DNA oligomer sequences attached to each DTHB. For instance, attaching four different DNA sequences to TAv creates heterotetravalent DTHBs (DTHB^ABCD^ in Fig. 2), while attaching identical DNA sequences forms isotetravalent DTHBs (DTHB^EEEE^ in Fig. 5). It is also possible to create tetravalent structures where only two or three distinct DNA sequences are binding-active, thereby forming what we refer to as partially heterotetravalent DTHBs (Fig. 2–4).

**Fig. 2:**
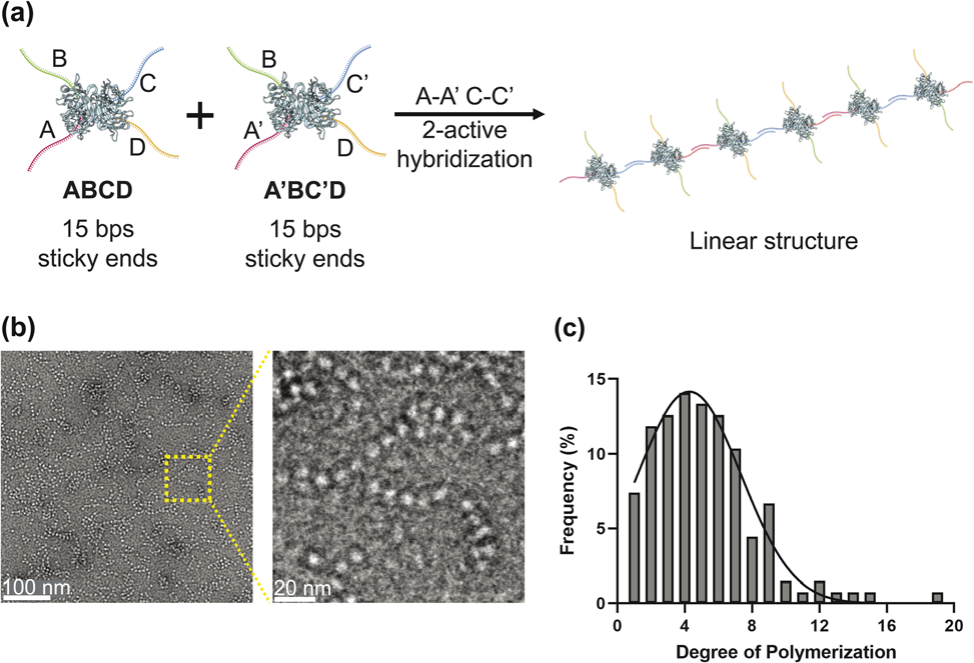
(a) Schematic illustration of self-assembled linear nanostructures formed *via* two-active DNA hybridization of DTHBs. Two building blocks were polymerized through hybridization of two complementary DNA pairs (A–A′, C–C′). (b) Negatively stained TEM images of the resulting linear nanostructures in a TAE buffer, and (c) degree of polymerization analysis indicating the transition to extended linear assemblies.

**Fig. 3:**
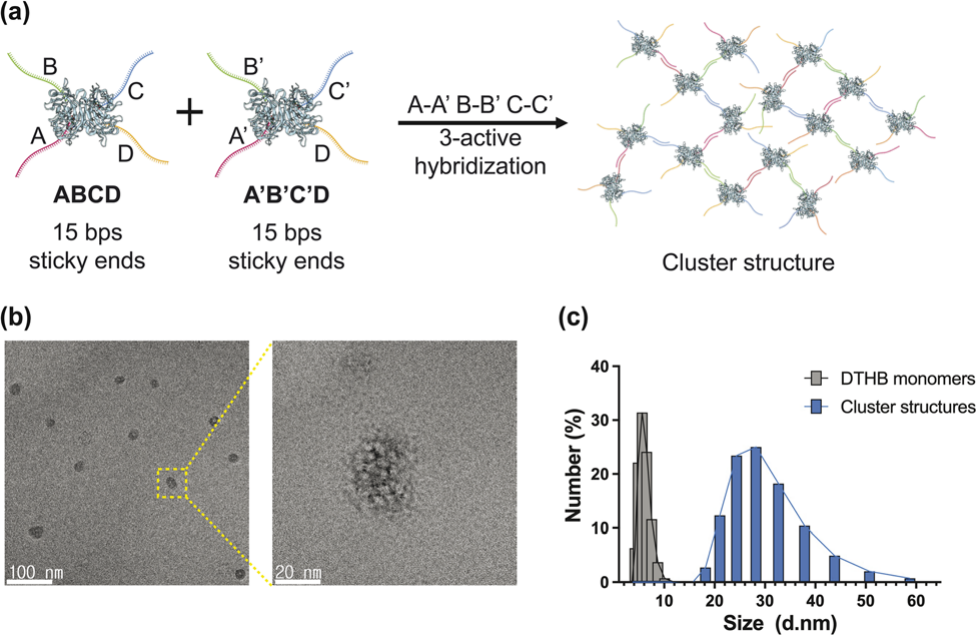
(a) Schematic illustration of cluster nanostructure assembly *via* three-active DNA hybridization of DTHBs. Two DTHBs were polymerized through hybridization of three complementary DNA pairs (A–A′, B–B′, C–C′). (b) Negatively stained TEM images of the resulting spherical cluster nanostructure and its (c) size distribution analysis measured by dynamic light scattering (DLS).

**Fig. 4:**
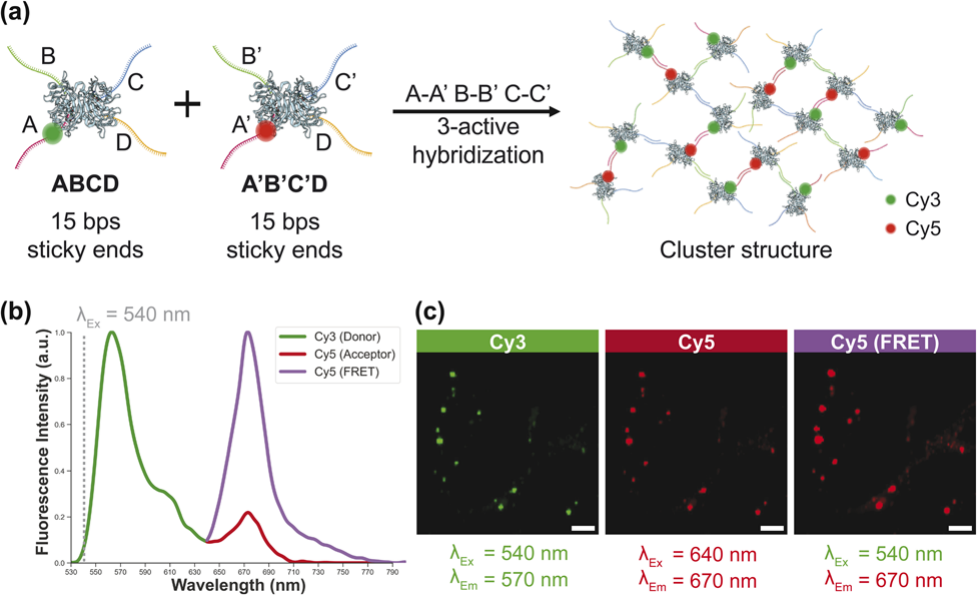
(a) Schematic illustration of clustered nanostructure assembly using Cy3-and Cy5-labeled DTHBs. Donor-labeled (Cy3) and acceptor-labeled (Cy5) DTHBs were assembled through complementary three-site active hybridization (A–A′, B–B′, C–C′). (b) Fluorescence emission spectra showing Cy3 (donor), Cy5 (acceptor), and Cy5 (FRET) signals all upon excitation at 540 nm, indicating efficient energy transfer between closely spaced Cy3 and Cy5 pairs within assembled clusters. (c) Confocal laser scanning microscopy (CLSM) images of dye-labeled nanoclusters on a glass substrate, collected under three different excitation/emission conditions. The Cy5 channel was imaged with excitation at 640 nm, while the FRET signal was detected *via* Cy5 emission upon donor excitation at 540 nm. Scale bars = 10 μm.

We established three types of resultant nanostructures, such as linear, cluster, and lattice forms, and defined their structural characteristics. Linear structures were formed by introducing two DTHBs that have two complementary ssDNAs (A–A′, B–B′) in each DTHB, as shown in Fig. 2(a). We mixed DTHB^AC^ and its complementary DTHB^A^′^C^′ in TAE-Mg^2+^ 12.5 mM buffer at the same molar ratio, followed by 3 hours of incubation at room temperature to encourage monomers to polymerize alternately. Multiple linear chains were observed in negatively stained transmission electron microscopy (TEM) images, confirming that the structure was built as we programmed (Fig. 2(b)). This was significantly different from the DTHB monomers (Fig. S3). TEM images also revealed that the linear nanoarchitecture's degree of polymerization (DP) peaked at 4–5 (SI S5), consistent with gel electrophoresis results (Fig. 2(c), S4 and S5). The surface charge of our building block was calculated using dynamic light scattering (DLS) in PBS buffer at pH 7.4, resulting in a zeta potential of −10.9 ± 0.5 mV. While we did not test the effect of zeta potential on subsequent supramolecular assembly, we think a degree of polymerization is influenced by its inherent negative potential.

Similarly, we combined two DTHBs (DTHB^ABC^ and DTHB^A′B′C′^) with three complementary ssDNAs (A–A′, B–B′, C–C′) in the same molecular ratio for the assembly of cluster supramolecular architectures, as shown in Fig. 3(a). TEM images illustrated the spherical morphology of the assembled structures, which we termed “cluster” structures (Fig. 3(b)). DLS measurements showed the average size of the spherical supramolecular structure to be 29.50 nm, compared to 5.95 nm for the DTHB monomer alone (Fig. 3(c)).

To further characterize cluster structures, we applied the Förster resonance energy transfer (FRET), a distance-dependent process in which energy is transferred from a donor fluorophore to an acceptor fluorophore. FRET provides a method to estimate nanoscale distances between labeled sites.^21^ In our system, Cy3 (donor) and Cy5 (acceptor) dyes were conjugated to the 5′ ends of biotin-modified DNA oligomers on the DTHBs (Fig. 4(a)). After assembling the DTHBs into cluster supramolecular structures, excitation at 540 nm wavelength was used to evaluate co-localization of Cy3 and Cy5 within the clusters. The close proximity of the dyes led to amplified Cy5 emission around 670 nm. We observed a 4.62-fold enhancement of Cy5 fluorescence in the presence of donor, yielding a FRET efficiency of 82%. Based on this, we calculated the distance between Cy3 to Cy5 to be 4.65 nm, matching the expected distance from the DNA linker length (SI S6). Including the protein size, the estimated center-to-center distance between DTHBs was ∼9.65 nm. Given that the DNA used in this experiment is 15 bp, this result aligns well with our prediction. Confocal laser scanning microscopy (CLSM) imaging further confirmed the FRET phenomenon (Fig. 4(c)). Under 540 nm excitation, Cy3 fluorescence was detected in the Cy3 channel, while under 640 nm excitation, Cy5 fluorescence appeared in the Cy5 channel as expected. Notably, even with 540 nm excitation, we observed strong Cy5 channel intensity, indicating that energy absorbed by Cy3 was efficiently transferred to Cy5 by FRET.

Encouraged by these results, we further examined assembly using isotetravalent-DTHBs, in addition to the hetero-tetravalent-DTHBs used previously. Isotetravalent DTHBs were constructed with four identical sequenced DNA oligomers. Following the same methods for supramolecular nanostructure assembly, we created two types of building blocks with complementary sequences (DTHB^EEEE^ and DTHB^E′E′E′E′^) and mixed them to fabricate lattice structures. Given the three-dimensional tetrahedral structure of isotetravalent DTHBs due to the *D*_2_ symmetry of the TAv core, we hypothesized they could be assembled into specific lattice structures. Initially, we used 15 bps DNA attached DTHBs, similar to the previous building blocks. However, we observed that the highly ordered lattice structure did not fully fabricate with 15 bps-DNA building blocks. We inferred that the longer DNA strands hindered close-packing lattice formation.

To address this, we constructed the supramolecular structure using building blocks with shorter DNA strands (6 bps) (Fig. 5(a)). These were then subjected to a slow cooling step from 60 °C to room temperature overnight in 12.5 mM TAE-Mg^2+^ buffer to ensure lattice assembly. The tetrahedral symmetry of TAv and the vertex-to-vertex binding design of our DTHBs show the possibility of forming diamond-like lattices, but assembling into well-ordered diamond lattices is challenging, as the diamond lattice it forms is sensitive to bond and local heterogeneity and has low packing density.^22^ The free rotation of the building blocks can easily introduce defects, leading to a mixture of multiple local structures or disordered formations. Surprisingly, lattice structures using 6 bps DTHBs exhibited weak but ordered arrangements, while 15 bps DNA strands resulted in more disordered structures. Using optical microscopy and TEM analysis, we examined the resulting structures by their global morphologies and electron diffraction patterns. In the 15 bps DTHB lattices, we found no matching *q*/*q*0 ratios in the electron diffraction patterns with conventional lattice structures. Additionally, TEM images showed irregular particle distances, forming branch-like structures, indicating that not all four binding sites were hybridized (Fig. S7). In contrast, the 6 bps DTHB lattices annealed by the slow cooling process exhibited we noticed a crystal-like global morphology (Fig. S8). Indeed, the building blocks were observed to align in a distinct pattern (Fig. 5(b)). For further analysis, we utilized the selected area electron diffraction analysis method to determine the lattice configuration formed (SI S7). From the diffraction pattern in Fig. 5(c), we found that the majority of *q*/*q*0 peaks matched those of cubic diamond lattices.^23,24^ Although some peaks did not perfectly align, these results indicated that our isotetravalent DTHBs have the potential to form distinct lattices.

**Fig. 5:**
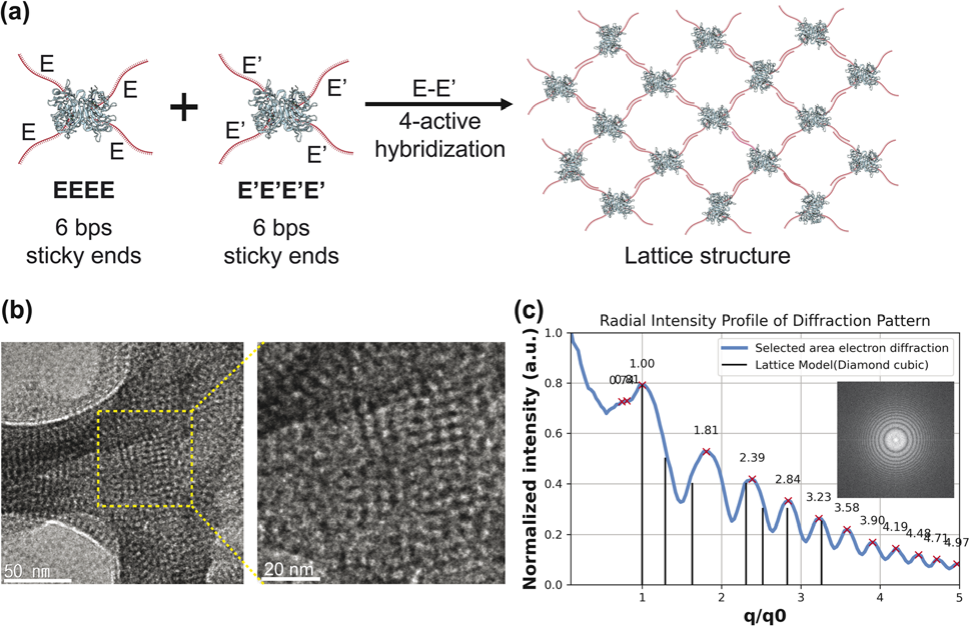
(a) Schematic illustration of the 6 bps-DNA conjugated isotetravalent DTHBs and the assembly of lattice structures. (b) TEM images of the resulting lattice structures. (c) Radial intensity profile obtained from selected area electron diffraction (SAED) of the lattice shown in (b), overlaid with the theoretical diffraction pattern for diamond cubic lattice. The inset shows the corresponding diffraction image.

We also tested the structures that would form using same 6 bps-DNA oligomer sequences with TAv protein, but with only two or three active binding sites. To create these building blocks, we mixed DNA with TAv at a 2 : 1 molar ratio. However, this approach resulted in the formation of a thin layer structure (Fig. S9), which was neither the 3D lattices nor the linear and clustered structures illustrated in Fig. 1. This finding explains that defects in DTHBs can lead to thin layer structures and demonstrates that at least four binding sites are required to form 3D crystalline lattices. Thus, allowing biotinylated DNA to effectively bind to the core protein is crucial. This also highlights the importance of programming the DTHBs to have a specific number of active DNA-binding sites to achieve controlled and sophisticated supramolecular structure assembly.

## Conclusions

In summary, we successfully fabricated DNA-protein hybrid building blocks (DTHBs) with TAv using four independently programmable biotinylated DNA oligomers. These building blocks do not require additional chemical modification in TAv's four binding domains, and they are formed simply by mixing proteins with specific DNA sequences. Our robust magnetic purification steps produced heterotetravalent DTHBs with four different DNAs attached to each of TAv's binding domains, while isotetravalent DTHBs, formed with identical DNA oligomers, did not require this magnetic purification step.

By constructing two types of building blocks that are designed to hybridize and control the number of active DNA binding sites, we successfully fabricated three distinct supramolecular structures in a predetermined manner. These structures were thoroughly characterized using various analytical techniques. We believe that controlling the hierarchical organization of supramolecular nanostructures composed of biocompatible molecules, such as DNA and proteins, holds great promise in materials science, particularly for biomedical applications, including drug delivery, therapeutics, and biosensors.^25–27^

## Author contributions

D.G. Lee: conceptualization, investigation, formal analysis, methodology, visualization, writing – original draft. Y.-Y. Kim: conceptualization, investigation, formal analysis, methodology. H. Yang: formal analysis. Y.-K. Song: conceptualization, funding acquisition, supervision, writing – review & editing.

## Conflicts of interest

There are no conflicts to declare.

## Supplementary Material

NA-OLF-D5NA00575B-s001

## Data Availability

The data supporting this article have been included as part of the SI. Supplementary information: Materials, methods, and additional results. See DOI: https://doi.org/10.1039/d5na00575b.
