# Supplementary material for: Engineering versatile supramolecular structures with tetravalent DNA-traptavidin building blocks
Source: Nanoscale Adv. 2025 Sep 2;7(20):6417–21. doi: 10.1039/d5na00575b (PMC12418037; doi:10.1039/d5na00575b)
Supplement: NA-007-D5NA00575B-s001 [file NA-007-D5NA00575B-s001.pdf]

Supplementary Information (SI) for Nanoscale Advances.
This journal is © The Royal Society of Chemistry 2025

Electronic Supplementary Information

Engineering Versatile Supramolecular Structures with Tetravalent DNA-Traptavidin Building Blocks

Dayoung Gloria Lee[†1,3], Young-Youb Kim[†1], Hoonil Yang[1], and Yoon-Kyu Song*[1,2]

[1]Graduate School of Convergence Science and Technology, Seoul National University, Seoul, 08826, Korea.

[2]Advanced Institute of Convergence Science and Technology, Suwon-si, Gyeonggi-do, 16229, Republic of Korea.

[3]Department of Chemical Engineering, Columbia University, New York, New York 10027, United States.

† D. Lee and Y–Y. Kim contributed equally to this work.

* Corresponding author: songyk@snu.ac.kr (Y.-K. Song)

Table of Contents

- **Materials and Methods**

Materials -- S3

Table S1. DNA sequences used in this study -- S3

Methods

1. Synthesis of DNA-modified magnetic beads. -- S4
2. Fabrication of tetravalent DNA-traptavidin building blocks. ------------------------------------ S4
3. Fabrication of identical-valent DNA-traptavidin building blocks. ------------------------------ S4
4. Supramolecular assembly using DNA-traptavidin building blocks ----------------------------- S5
5. Analysis of the degree of polymerization in linear structures. ----------------------------------- S5
6. Analysis of FRET efficiency and distance estimation between Cy3 and Cy5 in a cluster structure --- S5-6
7. Characterization of crystal structures using radial intensity profiles from electron diffraction patterns --- S6-7

- **Supporting figures**

Figure S1. The schematic image of stepwise magnetic separation method for purifying tetravalent building blocks. S7

Figure S2. Gel electrophoresis characterization...S8

Figure S3. Negatively stained TEM image of purified tetravalent DNA-traptavidin building blocks....S9

Figure S4. Negatively stained TEM image of linear supramolecular nanostructures...........................S9

Figure S5. (A) Band intensity analysis using Gel Doc Ez gel documentation system and (B) resultant degree of polymerization analysis...S10

Figure S6. Normalized fluorescence excitation and emission spectra of assembled cluster nanostructures. S10

Figure S7. Negatively stained TEM image of the assembly using 15bps isotetravalent DTHBs.........S12

Figure S8. (A) Schematic illustration of the lattice assembly using 6bps isotetravalent DTHBs. (B) Optical microscope images of the assembled lattices. (C) TEM images showing the structural morphology of the lattice...S13

Figure S9. (A) Schematic illustration of the lattice assembly using different valency DTHBs with 6-bps DNA, and the resulting morphology image captured by an optical microscope. (B) TEM images of the resulting structures at different magnifications..S14

- **References** -- S15

Materials and Methods

Materials

The chemical reagents [anhydrous Dimethyl sulfoxide (DMSO), AgNO3, (+)-sodium L-ascorbate, sodium dodecyl sulfate] were purchased from Sigma-Aldrich(St. Louis, MO, USA). HPLC-purified thiolated and biotin modified DNAs were purchased from IDT and Bioneer Inc. (USA, South Korea). The reduced DNAs were then purified through a desalting NAP-5 column (Sephadex G-25 medium, DNA grade) NANOpure H2O (18.0 MΩ), purified using a Milli-Q water purification system. Dynabeads M-270 amine and DynaMag™-2 magnet rack were purchased from Invitrogen. Succinimidyl-4-(p-maleimidophenyl)-butyrate (SMPB), Sulfosuccinimidyl acetate (Sulfo-NHS-acetate) and NHS-ester polyethylene glycol (PEG), MW 333 Da were purchased from Thermo Fisher Scientific (USA). The carbon coated copper grid (Ted Pella, Inc. Redding, CA, USA). Traptavidin protein was purchased from Kerafast, Inc.

Table S1. DNA sequences used in this study.

**Name**	**Sequence**
**Biotinylated ssDNA for synthesizing DNA-traptavidin building blocks**	
A	5’ - Biotin - A10 - CAA GTA CTC GGA ATG - 3’
B	5’ - Biotin - A10 - GTT ACT TCT GAT TAC - 3’
C	5’ - Biotin - A10 - ATC ATA GTG TTG GAG - 3’
D	5’ - Biotin - A10 - TGC CAT AGG AAT AGT - 3’
E	5’ - Biotin - GTC AGC - 3’
**Thiolated ssDNA for synthesizing DNA-modified magnetic beads**	
A’	5’ - SH - CAT TCC GAG TAC TTG - 3’
B’	5’ - SH - GTA ATC AGA AGT AAC - 3‘
C’	5’ - SH - CTC CAA CAC TAT GAT - 3‘
D’	5’ - SH - ACT ATT CCT ATG GCA - 3‘
E’	5’ - Biotin - GCT GAC - 3’
**Biotinylated ssDNA used in DNA-traptavidin building blocks for FRET analysis**	
A - Cy3	5’- Cy3 - iBiodT - CAA GTA CTC GGA ATG - 3’
A’- Cy5	5’- Cy5 - iBiodT - CAT TCC GAG TAC TTG - 3’

Methods

1. Synthesis of DNA-modified magnetic beads.

DNA-modified magnetic beads (MBs) were synthesized using amine-functionalized magnetic beads (Dynabeads M-270 Amine, Invitrogen) and single-strand DNAs followed by the same procedures reported in a previous study[1]. Amine-functionalized MBs were resuspended in 1 ml of anhydrous DMSO with 50 mg of SMPB, and incubated for 4 hours in a gentle continuous mixer. After discarding the supernatant and washing in a magnet rack (DynaMag™-2 magnet, Invitrogen) with a coupling buffer (0.1 M sodium phosphate buffer, pH 7.0 with 0.2 M NaCl) for two times, MBs were resuspended again in a coupling buffer containing 25 nmole of thiol-modified DNAs. Thiol-modified DNAs and MBs were incubated together with gentle mixing for 4 hours at room temperature (RT). Supernatant was discarded, and MBs were washed three times with coupling buffer and two times with passivation buffer (0.15 M sodium phosphate buffer, pH 8.0 with 0.15 M NaCl). MBs were resuspended in a passivation buffer containing 100 mg of sulfo-NHS-acetate for 1h at RT. After passivation, the MBs were washed three times with 20 ml passivation buffer, and resuspend to a final concentration of 10 mg/ml.

2. Fabrication of tetravalent DNA-traptavidin building blocks.

Four different biotinylated, 15 bps of single-stranded DNAs (125 pmol each) are randomly mixed with traptavidin (100 pmol) for 15 minutes at RT. Through biotin-avidin interaction, various resultant hybrid building blocks would be created considering the number of possible permutations. Therefore, four stepwise magnetic separation steps are necessary to purify tetravalent building blocks with four discrete DNAs among all randomly mixed samples. First, a DNA-modified magnetic bead hybridizes to a building block with a DNA strand that is complementary to the DNA sequence in the magnetic bead. Second, magnetic beads are captured by an external magnetic field in the magnet rack, and wash out the remaining extra DNA strands. Collected building blocks are washed three times with PBS solution to remove unbound residual DNAs. Finally, the bonds between the two strands of DNAs fall apart when we increased the temperature up to 60 ℃ for 5 min, so that the building blocks with the desired DNA sequence can be obtained. Those magnetic separation steps are repeated with other magnetic beads with other DNAs. Furthermore, to make dye conjugated building blocks, Cy3 and Cy5 dye conjugated DNA probes at the 5' end are added for binding to traptavidin. Next, dye conjugated multivalent building block complexes are purified in the identical magnetic separation process.

3. Fabrication of identical-valent DNA-traptavidin building blocks.

Four identical biotinylated, 6 bps of single-stranded DNAs (500 pmol) are mixed with traptavidin (100 pmol) for 15 minutes at RT. Through biotin-avidin interaction, identical-valent DNA-traptavidin hybrid building blocks would be created. Given that the building block has four identical DNA sequences, we did not try the magnetic separation technique here for purification.

4. Supramolecular assembly using DNA-traptavidin building blocks.

To demonstrate three different types of distinct supramolecular nanostructures using DNA-traptavidin building blocks (DTHBs), two complementary sets of DTHBs were incubated in Tris-acetate-EDTA-Mg^{2+} buffer (40mM Tris, 20mM acetic acid, 1mM EDTA, 12.5mM $MgCl_2$, pH 8.0). Linear supramolecular structures were assembled via combining two tetravalent DTHBs at an equimolar ratio of 1 pmole (10 nM, 100 μl), which have two complementary ssDNA linkers, A-A' and C-C'. After incubation at 60 °C for 10 min, cooled down to RT for 3 hours. Similarly, cluster supramolecular nanostructures were formed via combining two DTHBs at an equimolar ratio of 1 pmole (10 nM, 100 μl), which have three complementary ssDNA linkers (A-A', B-B', and C-C'). They were mixed at 60 °C for 10 min and at RT for 3 hours. Furthermore, two identical-valent DTHBs are mixed at an equimolar ratio of 200 pmole (2 μM, 100 μl) to construct lattice nanostructures followed by a slow cooling method from 60 °C to RT in a 2 L of boiled water containing Styrofoam box for 24 hours. After annealing, the resultant nanostructures were stored at 4 °C.

5. Analysis of the degree of polymerization in linear structures.

The degree of polymerization (DP) was determined by manually counting the number of clearly resolved protein monomers in each linear assembly, as observed in negatively stained TEM images[2]. A total of n = 135 chains were analyzed to calculate the average DP.

DP = number of monomers (proteins) per chain.

6. Analysis of FRET efficiency and distance estimation between Cy3 and Cy5 in a cluster structure.

Förster resonance energy transfer (FRET) is a powerful technique used to measure the distance between two fluorophores at the nanometer scale. We conducted a detailed analysis of the FRET efficiency and the donor-acceptor distance between Cy3 (donor) and Cy5 (acceptor) flurophores in a system using peak intensity values obtained from fluorescence spectra. The peak intensities at the acceptor's emission wavelength (670nm) were measured for three different conditions: the donor (Cy3) without the acceptor, the acceptor (Cy5) without the donor, and the donor with the acceptor.

The Förster radius (R_0) for the Cy3-Cy5 pair was taken as 6 nm from the previous study[3]. The fluorescnce intensities at 670 nm were as follows: the donor without the acceptor had an intensity of 0.00, the acceptor without the donor had an intensity of 0.21, and the donor with the acceptor had on intensity of 0.95. These values were used to calculate the enhancement factor, FRET efficiency, and the donor-acceptor distance.

The enhancement factor was calculated using the formula:

$$Enhancement\ factor = \frac{I_{DA} - I_{D0}}{I_{A0}}$$

where I_{DA} is the intensity of the donor with the acceptor, I_{D0} is the intensity oft he donor without the acceptor, and I_{A0} is the intensity oft he acceptor without the donor. Using the measured intensities, the enhancement factor was found to be 4.62.

The FRET efficiency (E) was then calculated using the enhancement factor:

$$E = \frac{Enhancement\ factor}{1 + Enhancement\ factor}$$

This yielded a FRET efficiency of 0.82, indicating a high efficiency of energy transfer from Cy3 to Cy5.

Finally, the distance (R) between the donor and acceptor was estimated using following formula:

$$R = R_0 \left(\frac{1-E}{E}\right)^{\frac{1}{6}}$$

With R_0= 6.0 nm and the calculated FRET efficiency (E = 0.82), the distance between the donor and acceptor was determined to be 4.65 nm. Considering that this distance represents the donor and acceptor distance in a hybridize dsDNA region, we can also estimate that the DTHB (DNA-traptavidin hybrid building block) to DTHB distance will be about 9.65 nm, including the protein size. Given that the DNA used in this experiment is 15 bp, this result correlates well with our prediction.

7. Characterization of crystal structures using radial intensity profiles from electron diffraction patterns.

Electron diffraction is a powerful tool for determining the atomic structure of materials. By analyzing the diffraction patterns obtained from Transmission Electron Microscopy (TEM), we can deduce the crystal structure and identify the lattice parameters of the sample. In this study, we used electron diffraction patterns to analyze the intensity distribution over reciprocal space distances, identify peaks corresponding to Bragg reflections, and compare the experimental results with multiple conventional lattice structures.

From the diffraction image, we extracted the radial intensity profile. The radial intensity profile was calculated by converting the diffraction image into radial coordinate system, centered on the primary beam spot. The intensity at each radial distance from the center was averaged to generate the radial intensity profile. The pixel distances were converted to reciprocal space distances (q-values) using a conversion factor calculated by ImageJ. The q-values are given by $q = \frac{2\pi}{d}$, where d is the inter-spacing.

To compare the theoretical Bragg reflections with our sample, we detected the peaks that exhibited high intensity profiles. Using the $\frac{q}{q0}$ ratios calculated in our experimental data, we compared them with the theoretical $\frac{q}{q0}$ ratios in conventional lattices, including simple-cubic (SC), body-centered-cubic (BCC), face-centered-cubic (FCC), hexagonal close-packed (HCP), diamond-cubic (DC), hexagonal diamond lattice.

The radial intensity profile obtained from the electron diffraction pattern showed distinct peaks corresponding to various Bragg reflections. These detected peaks were compared with the theoretical $\frac{q}{q0}$ ratios, and the

comparison revealed a close match between the experimental data and the theoretical predictions, confirming the presence of a diamond cubic structure in the sample.

Supplementary Figures

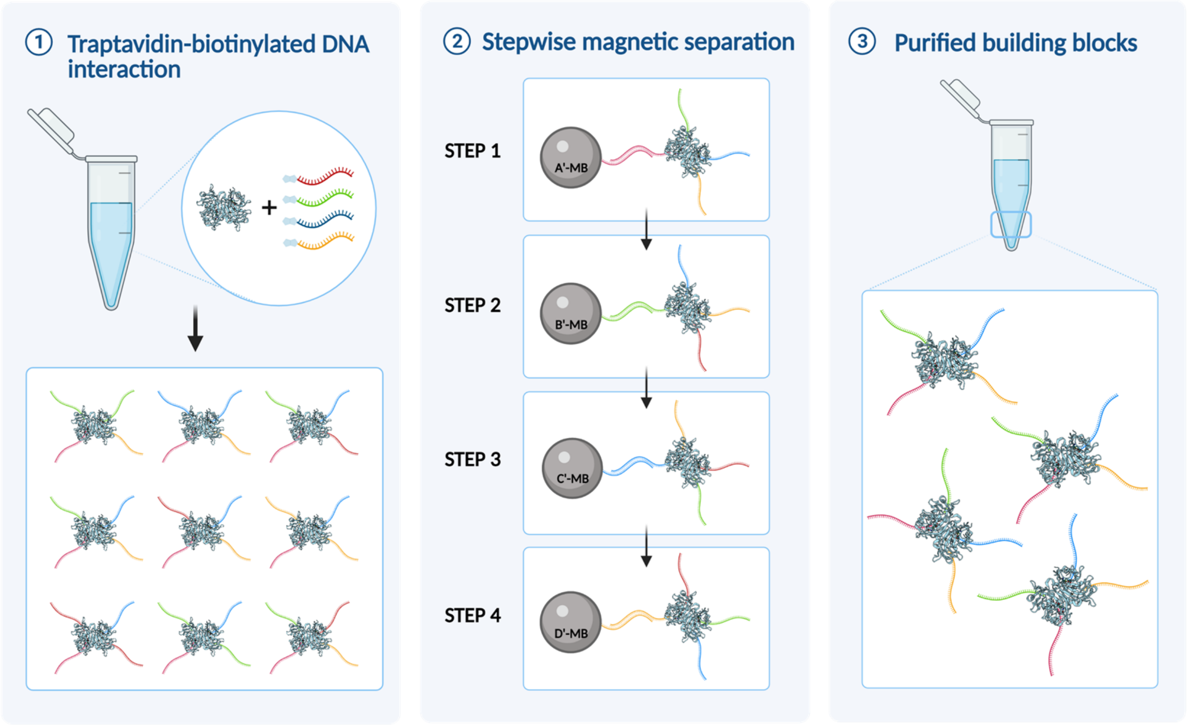

Figure S1. The schematic image of stepwise magnetic separation method for purifying tetravalent building blocks.

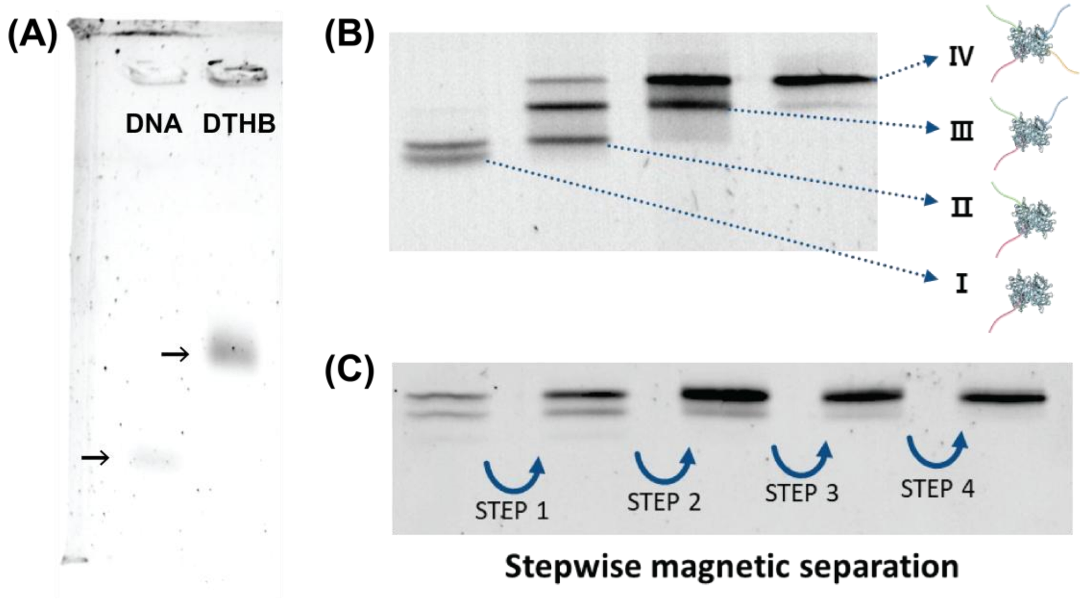

Figure S2. Gel electrophoresis characterization. (A) 2% agarose gel electrophoresis comparing DNA alone and DTHB samples at a traptavidin (TAv) : DNA molar ratio of 1:4. (B) Non-Denaturing 20% PAGE (37.5:1, acrylamide/bisacrylamide) images of DTHB in 0.5X TBE buffer at various traptavidin (TAv) : DNA molar ratios of 1:1, 1:2, and 1:4 through lane 1 to 3. Lane 4 illustrates the purified complex after four successive magnetic-separation steps. (C) PAGE images of the random mixture of TAv and biotinylated DNAs during four magnetic-separation steps. Lane 1 indicates the band image of the initially mixed sample at a traptavidin (TAv) : DNA molar ratio of 1:4. Lanes from 2 to 5 correspond to samples collected after each successive magnetic separation step. Gels were run at a constant voltage of 37.5 V/cm for 4 hours, stained with GelStar, and imaged using the Gel Doc EZ gel documentation system (Bio-Rad).

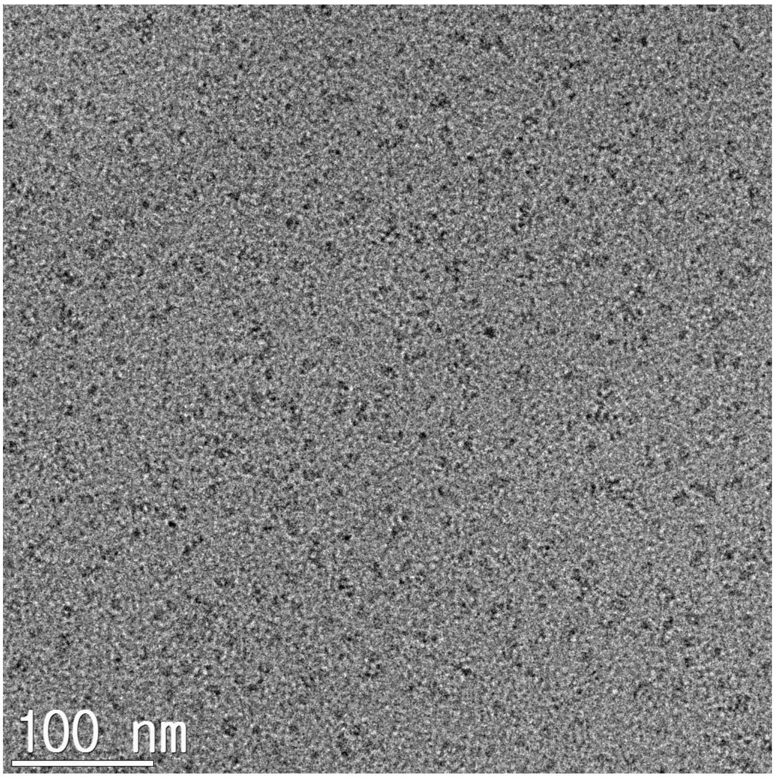

Figure S3. Negatively stained TEM image of purified tetravalent DNA-traptavidin building blocks.

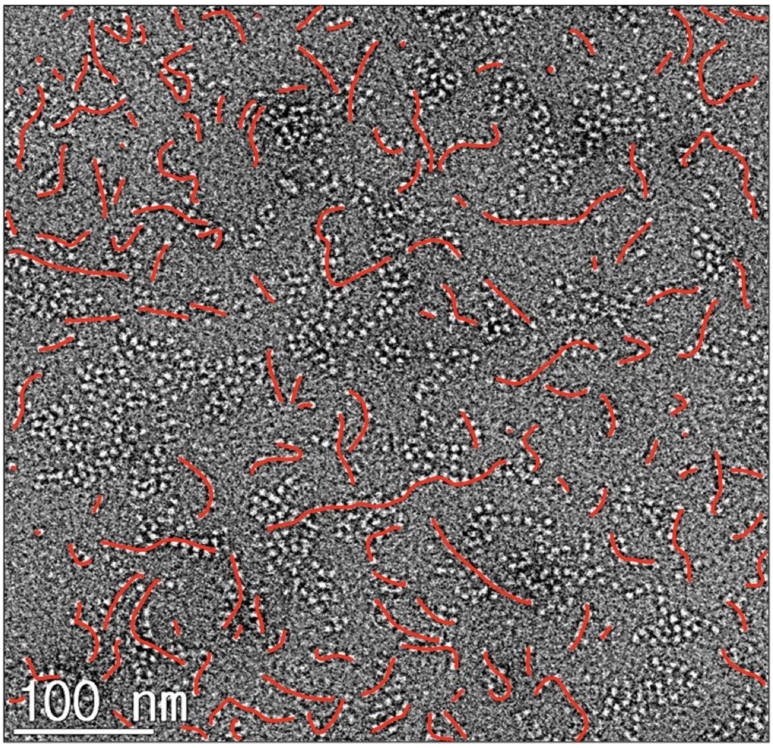

Figure S4. Negatively stained TEM image of linear supramolecular nanostructures. As indicated by the red line, the number of connected building block units on all the countable strands shown in this image were counted. The calculated degree of polymerization is shown in Figure 2(C).

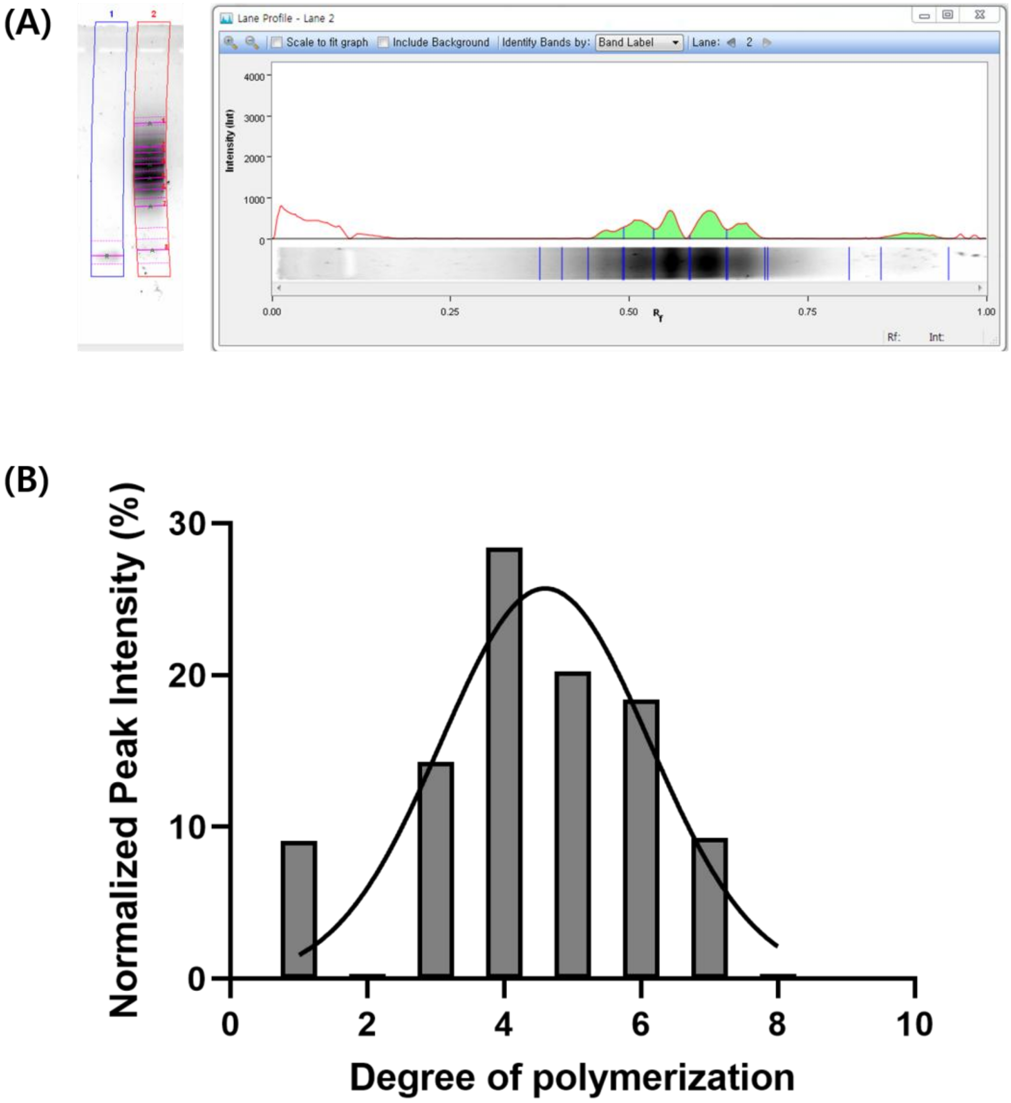

Figure S5. (A) Band intensity analysis using Gel Doc Ez gel documentation system. Lane 1: single DTHB. Lane 2: linear supramolecular structures formed by hybridization of two DTHBs with two active sticky end binding sites. Agarose (Sigma Aldrich) was added to 40 ml 1x TBE at the desired weight concentration (1 w/w %) and microwaved to dissolve the agarose completely. 1 µL GelStar was added to the gel and set at room temperature. 1 µM of samples 9 µL and 1 µL of 6X loading dye (invitrogen) were loaded. (B) Degree of polymerization (DP) analysis based on the TEM data shown in Figure S4. After normalizing peak intensities, the DP was calculated as described in the methods section 5.

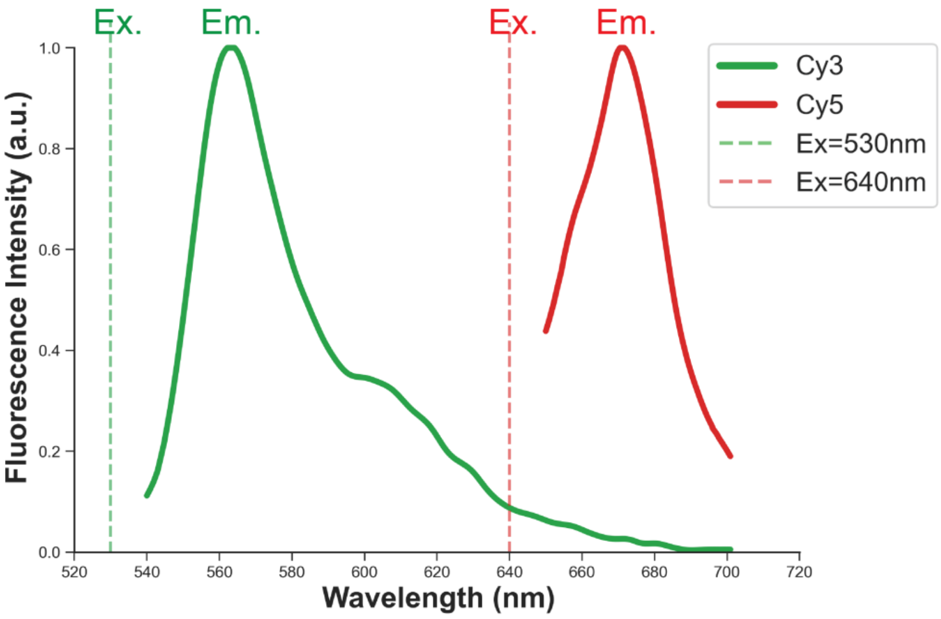

Figure S6. Normalized fluorescence excitation and emission spectra of assembled cluster nanostructures using two DTHBs labeled with Cy3 or Cy5, respectively. The green curve shows the emission profile of Cy3 following excitation at 530 nm (dashed green line), and the red curve corresponds to Cy5 emission upon excitation at 640 nm (dashed red line).

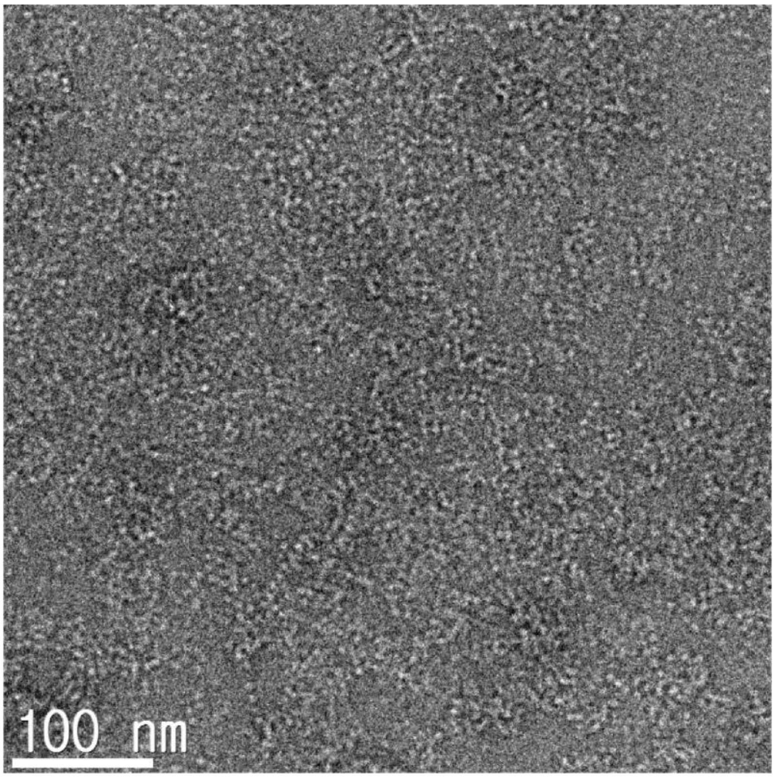

Figure S7. Negatively stained TEM image of the assembly using 15 bps of identical-valent DTHBs. The assembly was performed in the same method as used in Figure 5(B). The figure above confirms that the assembly using the 15 bps DNA modified identical-valent building blocks did not successfully construct a lattice structure.

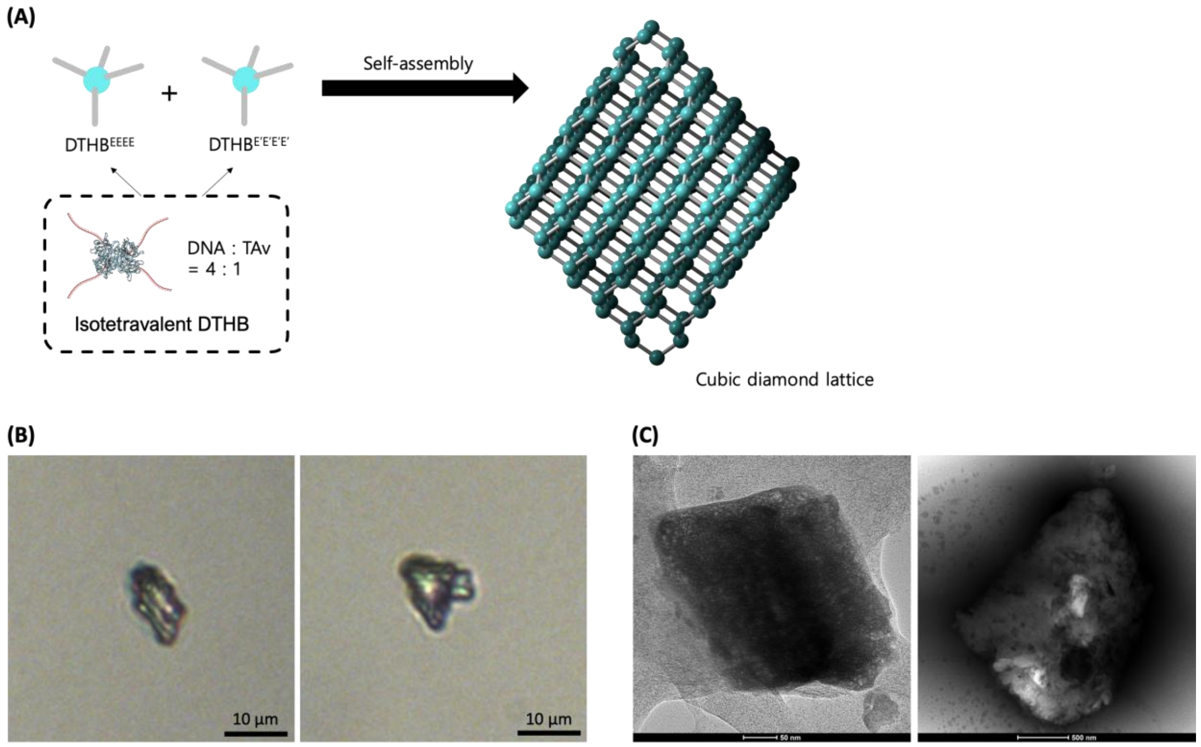

Figure S8. (A) Schematic illustration of the lattice assembly using 6-bps isotetravalent DTHBs. (B) Optical microscope images of the assembled lattices. Scale bar = 10 µm. (C) TEM images showing the structural morphology of the lattice.

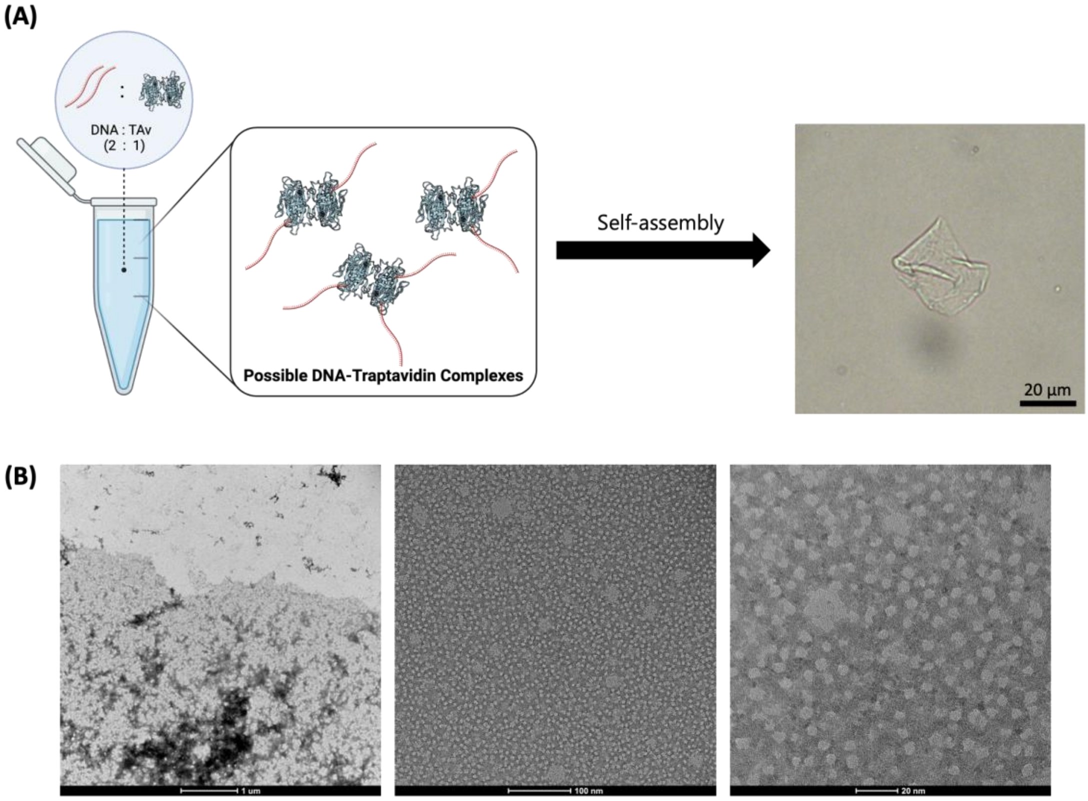

Figure S9. (A) Schematic illustration of the lattice assembly using different valency DTHBs with 6-bps DNA, and the resulting morphology image captured by an optical microscope. Scale bar = 10 µm. (B) TEM images of the resulting structures at different magnifications.

References

1 H. D. Hill and C. A. Mirkin, *Nat Protoc*, 2006, **1**, 324–336.

2 X. Lu, H. Fu, K.-C. Shih, F. Jia, Y. Sun, D. Wang, Y. Wang, S. Ekatan, M.-P. Nieh, Y. Lin and K. Zhang, *J. Am. Chem. Soc.*, 2020, **142**, 10297–10301.

3 S. Hohng, C. Joo and T. Ha, *Biophysical Journal*, 2004, **87**, 1328–1337.
